# Orbital apex syndrome: an unusual complication of herpes zoster ophthalmicus

**DOI:** 10.1186/s12879-015-0760-z

**Published:** 2015-01-31

**Authors:** Chun-Yuan Lee, Hung-Chin Tsai, Susan Shin-Jung Lee, Yao-Shen Chen

**Affiliations:** Division of Infectious Diseases, Department of Medicine, Kaohsiung Veterans General Hospital, Kaohsiung, Taiwan; Faculty of Medicine, School of Medicine, National Yang-Ming University, Taipei, Taiwan; Graduate Institute of Science Education and Environmental Education, National Kaohsiung Normal University, Kaohsiung, Taiwan

**Keywords:** Herpes zoster ophthalmicus, Orbital apex syndrome, Varicella zoster virus

## Abstract

**Background:**

Herpes zoster ophthalmicus is defined as herpes zoster involvement of the ophthalmic division of the trigeminal nerve. Ocular involvement occurs in 20–70% of patients with herpes zoster ophthalmicus and may include blepharitis, keratoconjunctivitis, iritis, scleritis, and acute retinal necrosis. Orbital apex syndrome is a rare but severe ocular complication of herpes zoster ophthalmicus. We present here the first reported case of herpes zoster ophthalmicus complicated by orbital apex syndrome in a patient from Taiwan.

**Case presentation:**

A 78-year-old man initially presented with patchy erythema and herpetiform vesicles on his left forehead and upper eyelid. He subsequently developed left-sided ocular complications including reduced visual acuity, anisocoria, ptosis, and complete ophthalmoplegia. Orbital magnetic resonance imaging (MRI) was performed on day 6 of admission to search for signs of the common causes of orbital apex syndrome such as hemorrhage, neoplasm, and cavernous sinus thrombosis. The MRI showed only orbital myositis and enhancement of the retro-orbital optic nerve sheath. The patient was diagnosed with herpes zoster ophthalmicus complicated by orbital apex syndrome. Although the ocular complications partially resolved after systemic antiviral therapy for 15 days and steroid therapy tapered over 12 weeks, there was residual limitation of abduction and paralysis of the left upper eyelid at follow-up at 180 days after the onset of symptoms. The orbital MRI findings at 180 days showed no significant changes compared with the MRI findings on day 6 of admission.

**Conclusions:**

Primary care physicians should be aware of this rare but potentially sight-threatening complication of herpes zoster ophthalmicus. The appropriate therapy for orbital apex syndrome due to herpes zoster ophthalmicus and the potential outcomes of this condition require further investigation.

## Background

Herpes zoster is a unilateral vesicular eruption with a dermatomal distribution that results from reactivation of latent varicella zoster virus (VZV) infection of the dorsal root ganglia. Herpes zoster ophthalmicus (HZO) is caused by reactivation of latent VZV infection in the trigeminal ganglion, involving the ophthalmic division of the trigeminal nerve (V1). The reported mean time to the onset of ocular involvement is 1.82 weeks (range, 1–4 weeks) after the start of skin lesions in the V1 dermatome [1]. Ocular involvement occurs in 20–70% of patients with HZO [1,2], and may include blepharitis, keratoconjunctivitis, iritis, scleritis, and acute retinal necrosis. Neurological complications are less frequent than ocular complications, and may include ophthalmoplegia, optic neuritis, ptosis, and (rarely) orbital apex syndrome (OAS) [3]. OAS involves dysfunction of V1, the oculomotor nerve (III), the trochlear nerve (IV), and the abducens nerve (VI), as well as dysfunction of the optic nerve (II). The causes of OAS include a variety of inflammatory, infectious, neoplastic, iatrogenic/traumatic, and vascular conditions. Aspergillosis and mucormycosis are the most common infectious causes of OAS, and VZV is rarely considered as a cause. We present here the first reported case of HZO complicated by OAS in a patient from Taiwan.

## Case presentation

A 78-year-old man presented to an ophthalmology clinic complaining of a gritty sensation in both eyes for several months, with exacerbation in the left eye over the past 2 weeks. He had a history of diabetes mellitus treated with oral hypoglycemic drugs and chronic obstructive pulmonary disease. He was diagnosed with background diabetic retinopathy. Examination using a Snellen chart showed a visual acuity of 6/12 in both eyes, meaning that he would need to approach to a distance of 6 meters to read letters that a person with normal acuity would be able to read at 12 meters. The patient noticed pain and swelling of his left eyelid at that time, and developed headache and vomiting 1 week later. Five days before admission, he developed patchy erythema with clustered vesicles with varying sizes on his left forehead and upper eyelid. On the day of admission, he developed drooping of the left upper eyelid, and was admitted to the infectious diseases ward with a diagnosis of HZO in the distribution of V1 on the left side, complicated by superficial punctate keratitis.

**Figure 1:**
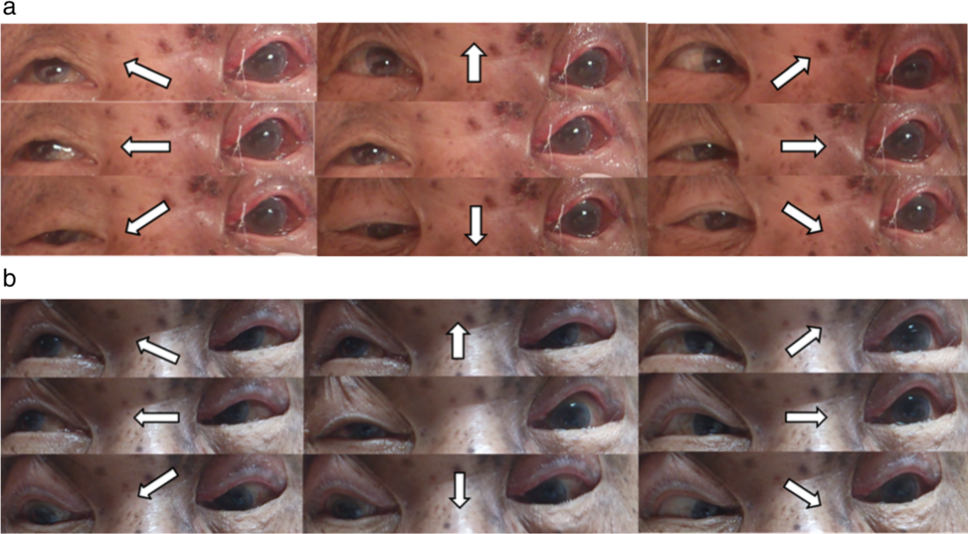
**Eye positions in each direction of gaze (arrows). a** On day 4 of admission, there was near-complete ophthalmoplegia and ptosis on the left side. **b** After 12 weeks of treatment, the ocular movements were markedly improved, except for abduction.

**Figure 2:**
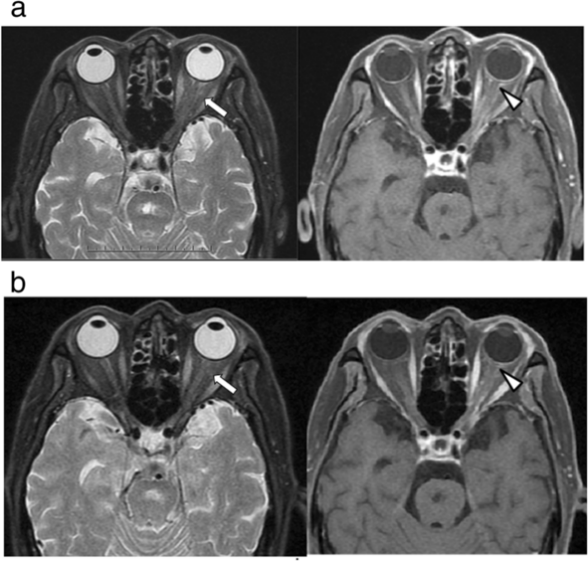
**Orbital MRI findings. a** On day 6 of admission, there was enhancement and swelling of the left extraocular muscles (arrows) on T2-weighted images; and mild enhancement of the left optic nerve sheath (arrowhead), left posterior orbital wall, and left orbital apex on contrast-enhanced T1-weighted images. **b** At 180 days after the onset of symptoms, there were no significant changes compared with the MRI findings on day 6 of admission.

The patient was initially treated with intravenous acyclovir (500 mg every 8 hours), topical antiviral ointment (acyclovir 3%), and non-steroidal anti-inflammatory drugs to relieve the pain caused by severe swelling and erythema of his left upper eyelid. On day 4 of admission, the inflammation of the left upper eyelid had resolved, but he was still unable to elevate the eyelid. Physical examination showed markedly crusted skin lesions in the distribution of V1 on the left side, with a positive Hutchinson’s sign and complete levator paralysis. Examination of the extraocular movements with the eyelids held open showed near-complete ophthalmoplegia on the left side (Figure 1a). His visual acuity was 6/12 on the right side and 3/60 on the left side. He had anisocoria with the left pupil, which was poorly reactive to light and accommodation, measuring up to 6 mm in diameter. He was diagnosed with OAS, and brain magnetic resonance imaging (MRI) on day 5 of admission showed swelling of the left periorbital tissues and left-sided exophthalmos, with no evidence of a retro-orbital space-occupying lesion. Orbital fat-suppressed MRI on day 6 of admission showed orbital myositis and enhancement of the optic nerve sheath (Figure 2a).

The patient was diagnosed with HZO complicated by OAS, and systemic steroid therapy was initiated on day 6 of admission. Adduction and supraduction of the left eye started to improve on day 3 after the initiation of steroid therapy. He received intravenous acyclovir (500 mg every 8 hours) for 15 days, and oral prednisolone (25 mg twice daily; >1 mg/kg per day) for 4 days followed by 12 weeks of prednisolone therapy that was tapered at monthly visits. The patient was discharged after completion of the acyclovir therapy. His visual acuity recovered to 6/12 on the right side and 6/30 on the left side, and his extraocular movements gradually improved, with residual limitation of abduction and paralysis of the left upper eyelid at the completion of the steroid therapy (Figure 1b). Follow-up at 180 days after the onset of symptoms showed persistent limitation of abduction and paralysis of the left upper eyelid. Repeat orbital MRI at 180 days showed no changes to the orbital lesions compared with the MRI findings on day 6 of admission (Figure 2b).

## Discussion

The present case illustrates a rare but severe complication of HZO, which was not initially recognized because of ptosis and severe dermatitis of the left upper eyelid. Orbital MRI excluded other common causes of OAS such as hemorrhage, neoplasm, and cavernous sinus thrombosis, and showed orbital myositis and enhancement of the retro-orbital optic nerve sheath. Adduction and supraduction of the left eye started to improve on day 3 after the initiation of steroid therapy. Although the patient received systemic acyclovir therapy for 15 days, and high-dose systemic steroid therapy that was tapered over 12 weeks, there was residual limitation of abduction and paralysis of the left upper eyelid at follow-up at 180 days after the onset of symptoms.

The diagnosis of herpes zoster is usually based on clinical findings. However, clinical differentiation between VZV and zosteriform herpes simplex virus lesions may be difficult. Laboratory investigations such as viral cultures, antigen detection, and molecular techniques can reliably differentiate between these two conditions. Some clinical findings such as the patterns of clustering and recurrence of vesicles can also help to differentiate between the two conditions. The vesicles are uniform in size in herpes simplex infection, and vary in size in VZV infection [4]. Only 4% of patients with VZV infection experience a second episode of vesicles, whereas zosteriform herpes simplex virus lesions tend to recur [5,6]. Although we did not perform laboratory investigations to detect VZV, we consider that our patient was more likely to have VZV lesions than zosteriform herpes simplex virus lesions because of the lack of recurrence during 6 months of follow-up and the varying sizes of vesicles in the clusters.

Mucormycosis and aspergillosis are the most common infectious causes of OAS, and should be suspected in patients with predisposing conditions such as diabetes mellitus, alcoholism, hematologic malignancy, and immunosuppression. The primary infection occurs in the paranasal sinuses, with direct invasion into the orbital cavity [7]. Another infectious cause of OAS is bacterial infection of the paranasal sinuses complicated by cavernous sinus thrombosis. The most commonly implicated bacteria are *Staphylococcus aureus*, *Streptococcus pneumoniae*, and other streptococci. Diagnosis of these infections is straightforward because of the clinical manifestations, host factors, and radiological findings. Reactivation of latent VZV infection rarely presents as OAS, and presentation with ocular muscle palsy is uncommon in HZO. In the Mayo Clinic series, 3 of 86 patients with HZO (3.4%) had extraocular muscle palsy, with cranial nerve IV dysfunction in 2 patients and cranial nerve VI dysfunction in 1 patient [8]. In the Moorfield Eye Hospital study, 133 of 1356 patients with HZO (9.8%) had extraocular muscle palsy, with cranial nerve III dysfunction in 4 of the patients [9]. The incidence of cranial nerve II dysfunction was even lower, occurring in only 0.4% of the patients in the Moorfield Eye Hospital study [9]. HZO complicated by OAS, which is also known as optic neuritis/neuropathy associated with complete ophthalmoplegia, was first reported by Ramsell [10]. To the best of our knowledge, this is the first reported case of HZO complicated by OAS in a patient from Taiwan. Most previously reported cases of OAS due to HZO were in patients aged over 60 years [10-14], and one case occurred in a 29-year-old woman with severe immunological dysfunction because of acquired immunodeficiency syndrome [15]. In most cases, OAS developed within 3 weeks of the onset of a herpetic skin eruption [10-14].

Although the optimal therapy for OAS due to HZO remains unclear, the current mainstay of treatment is combined administration of systemic acyclovir and steroids [11-15]. The effectiveness of acyclovir therapy may have been reduced in our patient because of the delay in initiation of treatment. The optimal time for initiation of treatment is within 72 hours of the onset of the rash [5], but our patient did not receive acyclovir until 5 days after the onset of patchy erythema with herpetiform vesicles. In addition, the role of systemic corticosteroid therapy remains controversial. In our patient, adduction and supraduction started to improve on day 3 after the initiation of steroid therapy, but further improvement of the extraocular movements and ptosis was minimal, with residual limitation of abduction and paralysis of the left upper eyelid after 12 weeks of systemic steroid therapy. Orbital MRI at 180 days after the onset of symptoms showed continued swelling of the left extraocular muscles and mild enhancement of the left optic nerve sheath. Residual limitation of extraocular movements was also reported in other cases [12,14]. The proportion of patients who achieve resolution of ptosis, ophthalmoplegia, and reduced visual acuity remains unknown. Complete or near-complete resolution of ophthalmoplegia due to HZO has been reported to occur in 76.5% of cases, and may take between 2 weeks and 1.5 years (mean, 4.4 months) [3].

The mechanisms by which optic neuropathy and extraocular muscle paralysis develop in patients with OAS due to HZO are not completely understood. Possible mechanisms include extensive inflammation around the posterior ciliary nerves and vessels with ocular ischemia [16], orbital soft tissue edema with direct compression of cranial nerves III, IV, and VI [17], and direct spread of VZV from cranial nerve V to cranial nerves III, IV, and VI [18]. Different mechanisms may result in different prognoses [9].

## Conclusions

OAS is a rare but severe complication of HZO. Primary care physicians should be aware of this complication and should monitor patients carefully during the first 3 weeks after the onset of HZO. Orbital MRI should be performed to exclude more common causes of OAS. Further studies are needed to evaluate the appropriate therapy for OAS due to HZO, and the potential outcomes of this rare complication.

## Consent

Written informed consent was obtained from the patient for publication of this Case report and any accompanying images. A copy of the written consent is available for review by the Editor of this journal.

## Ethics statement

This study was conducted in compliance with the guidelines of the Declaration of Helsinki, and was approved by the ethics committee of Kaohsiung Veterans General Hospital.

## Abbreviations

HZO: Herpes zoster ophthalmicus
MRI: Magnetic resonance imaging
OAS: Orbital apex syndrome
VZV: Varicella zoster virus
